# Exploring Rain as Source of Biological Control Agents for Fire Blight on Apple

**DOI:** 10.3389/fmicb.2020.00199

**Published:** 2020-02-14

**Authors:** Marco E. Mechan Llontop, Kelly Hurley, Long Tian, Vivian A. Bernal Galeano, Hans K. Wildschutte, Sasha C. Marine, Keith S. Yoder, Boris A. Vinatzer

**Affiliations:** ^1^School of Plant and Environmental Sciences, Virginia Tech, Blacksburg, VA, United States; ^2^Department of Biological Sciences, Bowling Green State University, Bowling Green, OH, United States; ^3^Department of Biochemistry, Virginia Tech, Blacksburg, VA, United States; ^4^Alson H. Smith Jr. Agricultural Research and Extension Center, Virginia Tech, Winchester, VA, United States

**Keywords:** rain, fire blight, biocontrol, genome-based identification, UV mutagenesis

## Abstract

Poor survival on plants can limit the efficacy of Biological Control Agents (BCAs) in the field. Yet bacteria survive in the atmosphere, despite their exposure to high solar radiation and extreme temperatures. If conditions in the atmosphere are similar to, or more extreme than, the environmental conditions on the plant surface, then precipitation may serve as a reservoir of robust BCAs. To test this hypothesis, two hundred and fifty-four rain-borne isolates were screened for *in vitro* inhibition of *Erwinia amylovora*, the causal agent of fire blight, as well as of other plant pathogenic bacteria, fungi and oomycetes. Two isolates showed strong activity against *E. amylovora* and other plant pathogenic bacteria, while other isolates showed activity against fungal and oomycete pathogens. Survival assays suggested that the two isolates that inhibited *E. amylovora* were able to survive on apple blossoms and branches similarly to *E. amylovora*. Pathogen population size and associated fire blight symptoms were significantly reduced when detached apple blossoms were treated with the two isolates before pathogen inoculation, however, disease reduction on attached blossoms within an orchard was inconsistent. Using whole genome sequencing, the isolates were identified as *Pantoea agglomerans* and *P. ananatis*, respectively. A UV-mutagenesis screen pointed to a phenazine antibiotic D-alanylgriseoluteic acid synthesis gene cluster as being at the base of the antimicrobial activity of the *P. agglomerans* isolate. Our work reveals the potential of precipitation as an under-explored source of BCAs, whole genome sequencing as an effective approach to precisely identify BCAs, and UV-mutagenesis as a technically simple screen to investigate the genetic basis of BCAs. More field trials are needed to determine the efficacy of the identified BCAs in fire blight control.

## Introduction

There has been a growing effort in scouting for bacteria and fungi to be deployed as biological control agents (BCAs) against crop pests (Jaffuel et al., 2019) and diseases (Durán et al., 2018). The motivation behind this effort includes the emergence of fungicide resistance in plant pathogenic fungi and antibiotic resistance in plant pathogenic bacteria, as well as an increase in consumer demand for crops produced without synthetic pesticides (Dean et al., 2012; Reganold and Wachter, 2016; Sundin and Wang, 2018).

One plant disease that is challenging to control is fire blight of apple (*Malus x domestica*) and pear (*Pyrus communis*) (Norelli et al., 2003). Since this bacterial disease was first reported in the United States in the 1870s, the causal agent, *Erwinia amylovora*, has spread to Europe, Asia, and Africa and New Zealand, causing significant economic losses (CABI, 2018). The main vectors of transmission are rainfall and pollinating insects. *E. amylovora* mainly invades plants through open blossoms and wounds and infects trees systemically through the vascular system (Thomson, 1985; Koczan et al., 2011). The first sign of disease consists of droplets of bacterial ooze on the surface of infected tissues. Blossoms and young fruits are later aborted, followed by necrosis and wilting of leaves and shoots on infected branches. In the worst case, the entire tree may die (Norelli et al., 2003).

The antibiotic streptomycin sulfate is generally an efficient method to control fire blight, but antibiotic use in crop production is illegal in many countries, and antibiotic-resistant strains have emerged in several apple and pear growing regions (Loper et al., 1991; McManus and Jones, 1994; Russo et al., 2008; Förster et al., 2015; Tancos et al., 2015). Therefore, BCAs for fire blight control have been explored for many years (Ishimaru et al., 1988). Currently available commercial products include: BlightBan^TM^ A506 [*Pseudomonas fluorescens* A506, isolated from leaves of pear trees (Wilson and Lindow, 1992)], BlightBan^TM^ C9-1 [*Pantoea vagans* C9-1, isolated from apple stem tissue (Ishimaru et al., 1988)], Serenade Optimum^TM^ (*Bacillus subtilis* QST713, isolated from soil), Double Nickel^TM^ (*Bacillus amyloliquefaciens* D747, isolated from soil), Biopro^TM^ (*Bacillus subtilis* BD170), Bloomtime Biological^TM^ [*Pantoea agglomerans* E325, isolated from apple blossoms (Pusey, 1999)], and Blossom Protect^TM^ (*Aureobasidium pullulans* strains DSM 14940 and DSM 14941, isolated from leaves of apple trees in 1989, Germany). Disease suppression by these BCAs is achieved by multiple modes of action including: the production of antimicrobial compounds (Ishimaru et al., 1988; Temple et al., 2004), colonization rates higher than those of the pathogen (Wilson and Lindow, 1994), competition for nutrients (Wilson and Lindow, 1992; Lindow, 1993), induction of plant defenses (Van Wees et al., 1997; Pieterse et al., 2014; Alamri et al., 2019), or a combination of mechanisms (Neeno-Eckwall et al., 2001).

A major hurdle to the introduction of commercial BCAs is their regulation. Precise identification and thorough characterization are necessary to exclude the potential for a BCA to cause disease in plants, animals, or humans. In fact, Bloomtime Biological^TM^ is not available in the European Union because of safety concerns in regard to the species *P. agglomerans*, which has been reported to occasionally cause human infections (Cruz et al., 2007; Büyükcam et al., 2018). Precise genome-based classification and identification methods, such as the Life Identification Number (LIN) system implemented in the LINbase web server (Tian et al., 2019), could aid in regulation of BCAs, but those methods have not been thoroughly explored within this context.

Another challenge with BCAs is that their efficacy under field conditions is more variable than synthetic pesticides since BCAs contain living organisms, whose survival on plant surfaces and/or internal tissues depends on environmental conditions (Sundin et al., 2009; Bonaterra et al., 2012). This is especially true for BCAs that are applied to aerial parts of plants, where conditions can change rapidly in regard to temperature, humidity, and solar radiation. In a previous study (Failor et al., 2017), we isolated 33,134 bacterial strains from precipitation, of which 1144 strains were found to be putative ice nucleation active strains. Later characterization determined that 551 of those strains (i) did not have ice nucleation activity and (ii) were members of plant-associated species within the genera *Bacillus*, *Pseudomonas*, and *Pantoea*. These plant-associated species are also known to include BCAs. Because these bacterial strains were isolated from precipitation, we hypothesized that they may resist environmental stresses (solar radiation, temperature, moisture, etc.) better than bacteria isolated from soil or plant tissues. Therefore, we screened 254 of these ice nucleation inactive rain-borne isolates for inhibition of *E. amylovora* and for their ability to persist on apple trees. The strongest inhibitors of *E. amylovora* growth *in vitro* were then tested under laboratory and field conditions to evaluate survival on apple branches and blossoms and for suppression of fire blight on apple blossoms. The most promising BACs were then identified to species using genome-based methods, including the LINbase Web server (Tian et al., 2019), and the genetic basis of the biocontrol activity for one of the bacteria was explored using a combination of UV-mutagenesis and genome sequencing.

## Materials and Methods

### *In vitro* Screening for Inhibition of *E. amylovora* Growth

A dual culture assay was used to detect antimicrobial activity in 254 rain-borne bacteria (listed in Supplementary Table S1) against two isolates of *Erwinia amylovora* from Northern Virginia (BAV5616 and BAV5617). The assay was performed in Petri dishes containing yeast extract dextrose agar NYDA (Nutrient agar 23 g l^–1^, dextrose 10 g l^–1^, yeast extract 5 g l^–1^). 100 μl of *E. amylovora* suspensions were spread on each plate and up to five 10 μl-droplets of suspension of rain-isolated bacteria were placed at equal distance from each other. In an initial screening, suspensions were made by adding a loop-full of rain-borne bacteria to 1 ml of sterile 50 mM potassium phosphate buffer (pH 7.0). In follow-up experiments, concentrations of putative BCAs were adjusted to an optical density of 0.1 at 600 nm (OD_600_). Suspensions of increasing concentration of *E. amylovora* were spread on plates (OD_600_ of 0.001, 0.01, 0.1, and 1, with the latter corresponding to a concentration of 3 × 10^9^ CFU ml^–1^). Plates were incubated at 28°C for 24 h. Antagonistic activity was quantified by the diameter of the inhibition zone forming around the rain-isolated bacteria. Rain-isolated bacteria that showed the most pronounced inhibition were also screened for activity against other economically important bacterial (10 isolates), fungal (5 isolates) and oomycete (1 isolate) plant pathogens (Table 2 and Supplementary Table S2) using similar protocols.

The two rain-isolated bacteria, BAV2934 and BAV3296, that showed the strongest inhibition of *E. amylovora in vitro* were chosen for further characterization. To facilitate re-isolation from plants, rifampicin-resistant mutants were selected for these strains and for *E. amylovora*. This was done by growing strains on NYDA solid medium containing 20 mg ml^–1^ of antibiotic. Colonies that grew at this concentration were then serially transferred to media with increasing antibiotic concentration (50, 100, 150, and 200 mg ml^–1^).

### Survival Assay Under Environmental Conditions

To compare the survival of BAV2934, BAV3296, and *E. amylovora* rifampicin-resistant mutant strains (BAV5616), branches of potted 5-year old apple trees cultivar “Golden delicious” were spray-inoculated to run-off at an OD_600_ of 0.1 and placed on the roof of a three-story research building. Apple trees were exposed to the outdoor environment for the duration of the experiment. The non-pathogenic *Escherichia coli* strain DH5-Alpha was included for comparison. Bacterial population sizes were determined on the day of inoculation, and 4, 8, and 12 days after inoculation by dilution-plating on NYDA containing 200 mg ml^–1^ rifampicin.

### Assay of Inhibitory Activity Against *E. amylovora* on Detached Apple Blossoms

Blossoms of apple cultivar “Golden Delicious” were detached at the first bloom stage (central blossom just opened) and maintained with the cut pedicel submerged in 10% sucrose solution. Potential BCAs were cultured on NYDA plates for 24 h at 28°C, and cell suspensions were prepared in 10 mM MgSO_4_ containing 0.01% of Silwet. BlightBan^TM^ A560 (active ingredient: *P. fluorescens* strain A560; NuFarm Americas, Burr Ridge, IL, United States) and FireWall^TM^ 17WP (active ingredient: streptomycin sulfate; AgroSource, NJ, United States) were diluted to 0.8 g l^–1^ water and 2 g l^–1^ water, respectively, following manufacturer’s instructions. A rifampicin-resistant mutant strain of *E. amylovora* was cultured on NYDA plates containing 200 mg ml^–1^ rifampicin for 24 h at 28°C, and a cell suspension was prepared in 10 mM MgSO_4_ and 0.01% of Silwet.

Six individual detached blossoms were pre-treated with one of the following treatments: bacterial suspension (BAV2934, BAV3296 or *E. coli* DH5-Alpha) at a concentration of 10^9^ CFU ml^–1^; 10 mM MgSO_4_ (mock treatment negative control), or a commercial product (BlightBan^TM^ A560 or FireWall^TM^ 17WP). Detached blossoms were pre-treated by dipping the blossoms into the bacterial suspension or commercial product for 10 s. Blossoms were then allowed to air-dry for 2 min, enclosed in a planting tray, and incubated at room temperature for 48 h. The experiment was repeated a total of four times.

Pre-treated blossoms were then inoculated with *E. amylovora* at 10^7^ CFU ml^–1^, enclosed in a planting tray, and incubated at room temperature for 72 h. Pathogen population sizes were determined in the receptacle and pedicel separately by grinding plant tissue followed by dilution-plating on NYDA supplemented with 200 mg ml^–1^ of rifampicin. Disease severity was calculated as fraction of the length of the necrotic pedicel divided by the total pedicel length.

### Disease Suppression Assays on Attached Blossoms in an Orchard

Field experiments were conducted in April 2018 and April 2019 in an experimental orchard at Virginia Tech’s Kentland Farm (McCoy, VA, United States). Dates of treatments and weather conditions are reported in Supplementary Table S4. Clusters of apple blossoms of the varieties “Golden Delicious,” “York Imperial,” “Rome Beauty” and “Empire” at the first bloom stage were spray-inoculated to the point of run off. Inoculations were performed during morning hours to avoid warm temperatures that may affect bacterial attachment and survival. Between 20 and 30 blossom clusters were randomly pre-treated using BAV2934 (10^9^ CFU ml^–1^), BAV3296 (10^9^ CFU ml^–1^), FireWall^TM^ 17WP (2 g l^–1^), and BlightBan^TM^ A506 (0.8 g diluted in 1 l) as described above. 10 mM MgSO_4_ was used as mock treatment negative control. In 2018, three “Golden Delicious” and three “York” trees located in different rows in an orchard were inoculated as replicates, with each tree receiving all treatments. In 2019, three “Golden Delicious” and two “Empire” and two “Rome” apple trees located in different rows in an orchard were inoculated as replicates, with each tree receiving all treatments. Pre-treated blossoms were allowed to air-dry before inoculation with *E. amylovora* (10^7^ CFU ml^–1^). Disease incidence for each treatment was evaluated as percentage of symptomatic clusters out of all clusters. All data were analyzed using JMP statistical software (Statistical Analysis Systems Institute, Cary, NC, United States). We performed a one-way ANOVA analysis of variance, using a significance level of 0.05, to test the null hypothesis that treatments do not differ in controlling fire blight. Tukey-Kramer HSD was used for all pairwise comparisons in our detached and attached blossoms assays. Graphs were plotted in RStudio version 1.1.456 (RStudio, 2015).

### UV Mutagenesis Screen to Identify the Molecular Basis of Growth Inhibition

To identify genes necessary for the suppression of *E. amylovora*, mutants of BAV2934 were generated by UV radiation following the protocol by Barrick (2014) with small modifications. Briefly, an overnight bacterial culture was resuspended in water at an OD_600_ of 0.1. 10 μl droplets were subjected to 10,000 μJ of UV radiation utilizing a UV Stratalinker (Stratagene UV Stratalinker 1800). UV treated cells were plated onto TSA medium and incubated for 24 h at 28°C. All single colonies were tested for inhibitory activity following the plate assay as described above. Any putative bacterial colony that lost inhibitory activity against *E. amylovora* was confirmed in subsequent tests.

### Genome Sequencing

Genomic DNA from strains BAV2934 and BAV3296 was extracted using the Genomic DNA purification kit (PUREGENE – Gentra Systems, United States) according to the manufacturer’s protocol. The DNA concentration and quality were evaluated by UV spectrophotometry (NanoDrop 1000, Thermo Fisher Scientific, United States) and visualized on a 1% agarose gel. DNA library preparation and Paired-end (PE150) sequencing was performed on the Illumina HiSeq 2500 at the Biocomplexity Institute at Virginia Tech (Blacksburg, VA, United States).

Isolate BAV2934 was also sequenced using Oxford Nanopore Technologies’ (Oxford, United Kingdom) MinION. A total of 3 μg of purified bacterial DNA was treated with RNase A 100 mg ml^–1^ (1 μl for 100 μl of gDNA) and the Short Read Eliminator Kit (Circulomics, Baltimore, MD, United States), according to the manufacturer’s protocol. The purified bacterial DNA was used to prepare a sequencing library with the 1D genomic DNA ligation kit SQK-LSK109 (Oxford Nanopore Technologies, United Kingdom). Sequencing was performed with a FLO-MIN106 (R 9.4.1) flow cell for 48 h.

### Genome Analysis

FastQC (Andrews, 2010) was used to assess sequencing reads for quality control. Reads were trimmed using Trimmomatic (Bolger et al., 2014) to remove adapter sequences and low quality reads (*Q* < 30). *De novo* assemblies were generated using SPAdes (Bankevich et al., 2012).

The fast5 files generated by MinION sequencing of the BAV2934 genome were base-called using guppy (version 3.3.2). A hybrid assembly using both Illumina and ONT reads was performed using Unicycler v0.4.7 (Wick et al., 2017) with default parameters. Bandage (Wick et al., 2015) was used to visualize the bacterial assembly graph. Prokka (Seemann, 2014) was used to annotate the assembled genome sequence.

Genomic DNA of five BAV2934 UV-induced mutants was pooled and sequenced using Illumina as described above. Mutations were identified by mapping reads against the BAV2934 hybrid assembly. In brief, a reference genome index was pre-built with HiSat2 (Kim et al., 2015). Illumina paired-end reads were aligned to the reference genome using SAMtools (Li et al., 2009) and VarScan (Koboldt et al., 2009) was used for calling variants selecting parameters for pooled samples (–min-coverage8 –min-var-freq 0.15 -*p*-value 0.05). Read coverage of each gene was determined using bbmap (Bushnell, 2014).

Assembled genome sequences in fasta format were used for strain identification at the LINbase website at linbase.org (Tian et al., 2019) using the “Identify using a genome sequence” function.

The antibiotic and Secondary Metabolite Analysis Shell [antiSMASH (Medema et al., 2011)], which is capable of identifying loci that cover the whole range of known secondary metabolite compound classes, was used to identify gene clusters in the wildtype strain BAV2934.

### Analysis of Blossom Microbiomes

“Golden Delicious” apple blossoms were inoculated in an orchard as described above using BAV2934 (10^7^ CFU ml^–1^). Non-inoculated blossoms served as negative control. Fifteen blossoms per treatment were collected into a clean Ziploc plastic bag on day 0 (day of inoculation), 5 days after inoculation, and 10 days after inoculation. Blossoms were immediately transported to the laboratory and processed. Culturable bacterial population sizes were determined on TSA by dilution-plating.

For the culture-independent microbiome analysis, 300 ml of sterile distilled water was added to each Ziploc bag containing the blossoms. Samples were sonicated for 10 min using a 1520 BRANSON sonicator (Branson Ultrasonics, Danbury, CT, United States). The liquid was then vacuum-filtrated through a 0.2 μm pore-size filter membrane (Supor^®^ 200 PES membrane Disk Filter, PALL Corporation, Port Washington, NY, United States). Genomic DNA extraction from filters was performed using the DNeasy PowerWater kit (Qiagen, Germantown, MD, United States) according to the manufacturer’s protocol. DNA concentration and quality were determined by NanoDrop 1000 (Thermo Fisher Scientific, Waltham, MA, United States) and visualization on a 1% agarose gel. Primers 799F (anti-chloroplast, 5′AACMGGATTAGATACCCKG3′) and 1115R (“universal,”5′AGGGTTGCGCTCGTTG3′) were used to amplify and sequence the V4 hypervariable region of the 16S rRNA gene. All steps from PCR to paired-end (2 × 300 bp) amplicon sequencing on the Illumina MiSeq platform were performed at Molecular Research LP (MR DNA^TM^, Shallowater, TX, United States). QIIME version 1.9.1 was used for amplicon data analysis (Caporaso et al., 2010). Briefly, quality filtered paired-end reads (phred quality scores above 30) were joined together in a single read. Operational taxonomic units (OTUs) were picked using the open-reference pipeline at 97% sequence similarity using UCLUST as the clustering tool and the SILVA database (Quast et al., 2013). All OTUs unassigned or assigned to mitochondria, chloroplast and Cyanobacteria were removed. Data was visualized in RStudio version 1.1.456 using the Phyloseq 1.19.1 (McMurdie and Holmes, 2013) and ggplot2 2.2.1 packages.

## Results

### Rain-Borne Bacteria Inhibit *Erwinia amylovora* and Other Plant Pathogens *in vitro*

**FIGURE 1:**
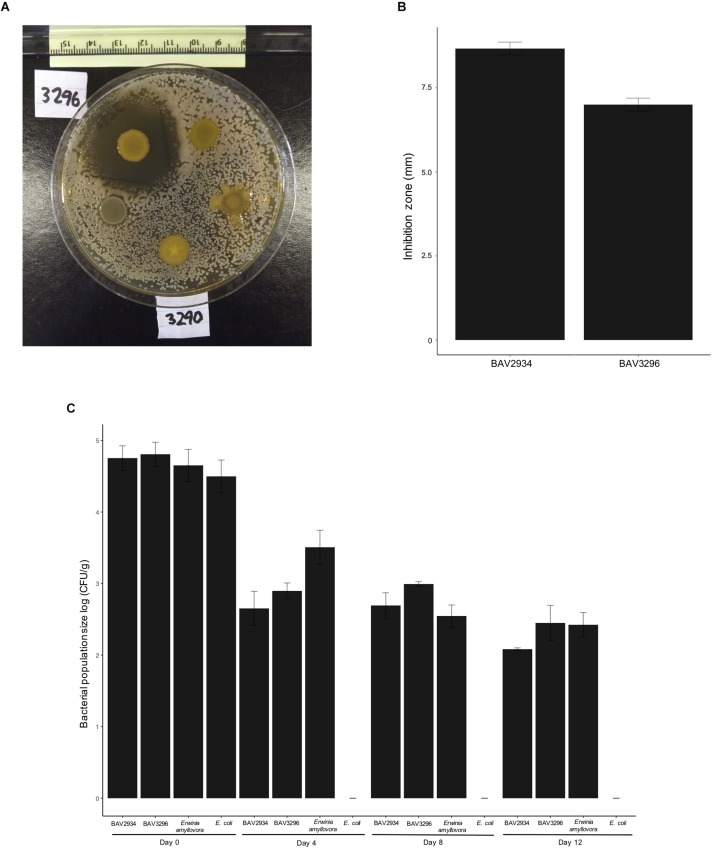
Inhibitory activity of rain-isolated bacteria against *E. amylovora* represented by **(A)** example of the halo inhibition assay showing the inhibition zone produced by strain BAV3296, **(B)** average size of the inhibition zone induced by strains BAV2934 and BAV3296, and **(C)** survival, measured as population size, of strains BAV2934 and BAV3296 compared with *E. amylovora* on detached apple branches under environmental conditions for up to 12 days. An *Escherichia coli* strain was included as control.

Nine out of 254 tested rain-isolated bacteria (Supplementary Table S1) displayed an inhibitory effect against *E. amylovora* during initial *in vitro* dual culture screening (Figure 1A). These nine bacteria had been previously identified (Failor et al., 2017) by 16S rRNA sequencing as members of the genera *Pseudomonas*, *Pantoea*, and *Bacillus* (Table 1). A more stringent assay using higher concentrations of *E. amylovora* allowed us to identify two isolates (BAV2934 and BAV3296) from the genus *Pantoea* with the highest inhibitory effect, as measured by the diameter of the inhibition zone that they induced (Figure 1B). BAV2934 and BAV3296 were chosen for further characterization.

**TABLE 1:** Identity of nine rain-isolated bacteria that showed antagonistic activity against *E. amylovora* in the initial inhibition screen.

Isolate	Source of isolation	Date of isolation	Location of rain collection	16S rRNA accession number	16S rRNA-based affiliation
BAV 2493	Rain	3/12/13	Private home, Blacksburg, VA, United States	KC901542.1	*Pseudomonas* sp.
BAV 2502	Rain	3/13/13	Private home, Blacksburg, VA, United States	KF956697.1	*Bacillus* sp.
BAV 2572	Rain	4/22/13	Kentland Farm, Blacksburg, VA, United States	NA^1^	*Pseudomonas* sp.
BAV 2934	Rain	5/15/13	Kentland Farm, Blacksburg, VA, United States	KT580675.1	*Pantoea* sp.
BAV 3049	Rain	6/26/13	Kentland Farm, Blacksburg, VA, United States	JX566609.1	*Pantoea* sp.
BAV 3226	Rain	9/26/13	Latham Hall, Blacksburg, VA, United States	KJ831070.1	*Pseudomonas* sp.
BAV 3280	Rain	9/28/13	Latham Hall, Blacksburg, VA, United States	NA	*Pseudomonas* sp.
BAV 3296	Rain	9/29/13	Latham Hall, Blacksburg, VA, United States	KR296701.1	*Pantoea ananatis*
BAV 4579	Rain	3/10/13	Private home, Blacksburg, VA, United States	KC901542.1	*Pseudomonas* sp.

*^1^These strains were identified based on the presence of a *Pseudomonas*-specific PCR product.*

**TABLE 2:** Inhibition assay of rain-isolated bacteria against bacterial, fungal and oomycete plant pathogens **(Part 1)**.

Rain isolated bacteria		*Xanthomonas* sp.	*Xanthomonas* sp.	*Pto* DC3000	*Pto* K40	*Pma* ES4326	*A. citrulli* AAC00-1	*Robbsia andropogonis*	*A. tumefaciens*
BAV2493	*Pseudomonas* sp.	–	++	–	–	–	+	–	++
BAV2502	*Bacillus* sp.	–	–	–	–	++	+	+	++
BAV2572	*Pseudomonas* sp.	+	+	+	+	+	–	–	+
BAV2934	*P. agglomerans*	+	++	+	+	+	–	+	+
BAV3049	*Pantoea* sp.	–	–	–	–	++	+	+	+++
BAV3226	*Pseudomonas* sp.	+++	+++	++	++	++	+++	+++	+++
BAV3280	*Pseudomonas* sp.	+++	+	++	++	++	++	++	+
BAV3296	*Pantoea ananatis.*	+++	+++	++	+	++	++	+++	+++
BAV4579	*Pseudomonas* sp.	+	+	–	–	–	+	–	++

**TABLE 2a:** Inhibition assay of rain-isolated bacteria against bacterial, fungal and oomycete plant pathogens **(Part 2)**.

Rain isolated bacteria		*Phytophthora capsici*	*Botrytis cinerea* 132	*Botrytis cinerea* 156	*Botrytis cinerea* 110/P6	*Colletotrichum acetatum* C15	*Colletotrichum* sp. C2
BAV2493	*Pseudomonas* sp.	–	–	–	–	–	–
BAV2502	*Bacillus* sp.	–	–	–	–	–	–
BAV2572	*Pseudomonas* sp.	+	–	–	–	–	–
BAV2934	*P. agglomerans*	–	–	–	–	–	–
BAV3049	*Pantoea* sp.	–	–	–	–	–	–
BAV3226	*Pseudomonas* sp.	+++	+++	+++	++	++	++
BAV3280	*Pseudomonas* sp.	+++	+++	+++	++	++	++
BAV3296	*Pantoea ananatis.*	–	–	–	–	–	–
BAV4579	*Pseudomonas* sp.	++	–	–	–	–	–

*+ = halo inhibition from 0.1–5 mm, ++ = halo inhibition from 5.5–10 mm, +++ = halo inhibition > 10 mm.*

We also evaluated the inhibitory effect of the nine bacteria that had shown activity against *E. amylovora* for activity against additional bacterial, fungal, and oomycete plant pathogens. BAV2934 and BAV3296 showed strong activity against the majority of the tested bacterial plant pathogens (including species of *Xanthomonas*, *Pseudomonas*, and *Ralstonia*), but not against fungal and oomycete pathogens, while the *Pseudomonas* strains BAV3226 and BAV3280 were most effective against fungal and oomycete pathogens (see a summary in Table 2 and quantitative results in Supplementary Table S2).

### Rain-Borne Bacteria Colonize and Survive Apple Trees Similar to *E. amylovora*

Survival, measured as bacterial population size over time, of *Pantoea* strains BAV2934 and BAV3296 and rifampicin-resistant *E. amylovora* BAV5616 was determined on detached apple branches placed on the top of our 3-story research building in three independent experiments performed on the following dates: from November 18th to 30th, 2016, from February 13th to 25th, 2017, and from March 24th to April 5th, 2017. Minimum and maximum temperatures during the three experiments ranged from −6 to 18°C, −7 to 18°C, 0 to 19°C, respectively. Apple branches in November 2016 and March 2017 were exposed to rain and light snow (details on weather conditions during the experiments are reported in Supplementary Table S3). Results for the experiment performed in February 2017 are shown in Figure 1C and results for experiments in November 2016 and March 2017 are shown in Supplementary Figure S1. In all three experiments, survival of BAV2934 and BAV3296 was comparable to *E. amylovora* after 12 days under environmental conditions (*p*-value 0.0001 ANOVA, Tukey HDS). The main difference between experiments was that in March 2017 the bacterial population size remained the same after 4 days post-inoculation, in contrast to the other two experiments in which the bacterial population size decreased 2-fold by day 4 post-inoculation.

**FIGURE 2:**
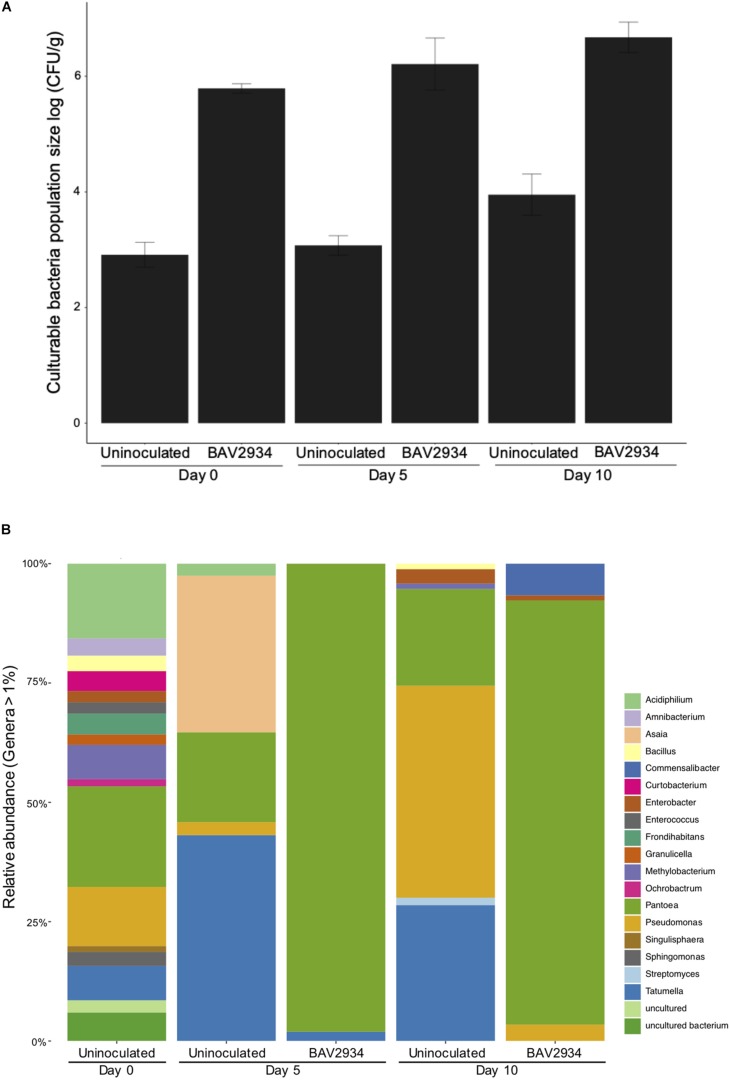
Survival of rain-isolated *P. agglomerans* BAV2934 on apple blossoms in an orchard. **(A)** Culturable bacteria, and **(B)** Relative abundance of bacterial taxa at the genus level (16S rRNA gene) present in blossoms up to 10 days after inoculation with BAV2934.

Population size of BAV2934 was also evaluated on apple blossoms at Kentland Farm in spring 2018 at 0, 5, and 10 days post-inoculation (April 25, April 30, and May 5 of 2018). Minimum and maximum temperatures during this experiment ranged from 0 to 27°C (Supplementary Table S4). In this case, the total bacterial population size was analyzed in combination with the relative abundance of BAV2934, as determined by culture-independent microbiome analysis. The total culturable bacterial population size on blossoms inoculated with BAV2934 was three-fold higher at 0, 5 and, 10 days post-inoculation, compared to the non-inoculated controls (Figure 2A). The relative abundance of BAV2934 was 95 and 85% at 5 days and 10 days post-inoculation, respectively (Figure 2B).

### Rain-Borne Bacteria Control Fire Blight on Detached Apple Blossoms

**FIGURE 3:**
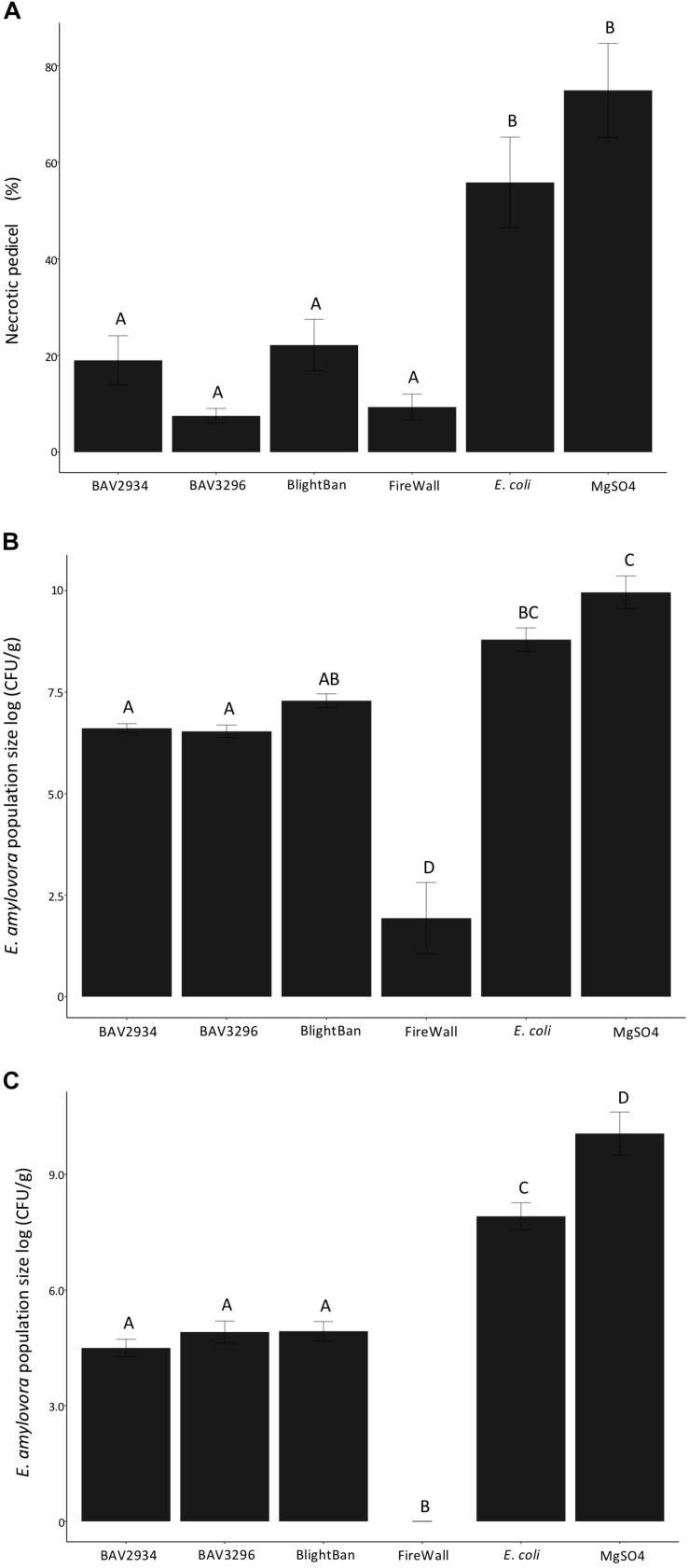
Antagonistic effect of rain-isolated bacteria BAV2934 and BAV3296 against *E. amylovora* on detached blossoms under laboratory conditions. **(A)** Disease severity measured by the necrotic tissue caused by *E. amylovora* invasion on the pedicel, **(B)** Pathogen population size in the receptacle, and **(C)** Pathogen population size in the pedicel 7 days after pre-treatment with either rain-isolated bacteria or commercial products.

Fire blight disease severity measured as a fraction of the length of necrosis along the pedicel divided the total pedicel length was significantly reduced by pre-treatments of blossoms with either BAV2934 (approximately 20% of necrotic pedicel) or BAV3296 (approximately 10% of necrotic pedicel), as compared to *E. coli* (60% of necrotic pedicel) and the MgSO4 mock treatment negative control (70% of necrotic pedicel) (*p*-value 0.0001, ANOVA, Tukey HDS). Treatments with the commercial products BlightBan^TM^ A506 (approximately 10% of necrotic pedicel) and FireWall^TM^ (approximately 5% of necrotic pedicel) were similarly effective with disease being less severe than the mock treatment for both of them (Figure 3A).

*Erwinia amylovora* population size in the receptacle and the pedicel was also significantly reduced by pre-treatment with either BAV2934 or BAV3296, compared to *E. coli* and MgSO_4_ (*p*-value 0.0001 ANOVA, Tukey HDS). In the receptacle, for example, the *E. amylovora* population was reduced by over 2-fold on blossoms treated with BAV2934 and BAV3296, compared to blossoms treated with *E. coli* or MgSO_4_ (*p*-value 0.0001 ANOVA, Tukey HDS). Similar pathogen population sizes were found in the receptacles of blossoms treated with BlightBan^TM^. However, BlightBan^TM^ was not as effective as FireWall^TM^, which suppressed pathogen growth almost completely (Figure 3B). In the pedicel, the effect of BAV2934 or BAV3296 was similar to BlightBan^TM^, in terms of *E. amylovora* population reduction. FireWall^TM^ again provided the most protection (Figure 3C).

### Rain-Borne Bacteria Show Inconsistent Results in Controlling Fire Blight in an Apple Orchard

Disease incidence of *E. amylovora*–inoculated apple blossoms pre-treated with BAV2934 and BAV3296 was compared with disease incidence following pre-treatments with commercial products BlightBan^TM^ A506 and FireWall^TM^ or a mock treatment negative control (10 mM MgSO_4_) in spring 2018 and spring 2019. Disease incidence was calculated as percentage of blossom clusters with fire blight symptoms out of the total number of clusters used in each treatment.

**FIGURE 4:**
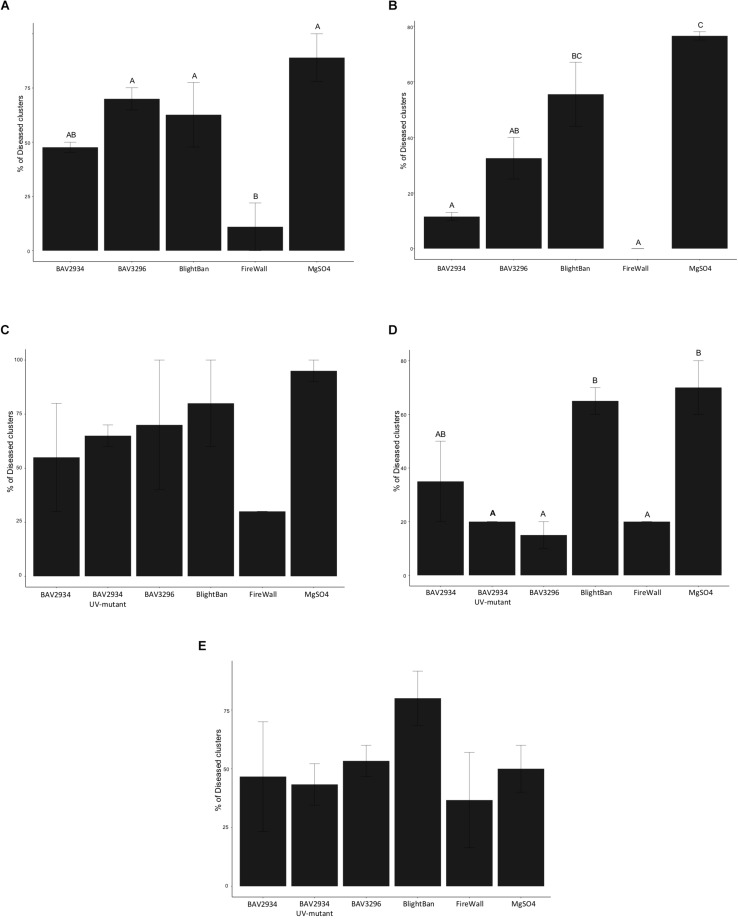
Fire blight control on blossoms in an apple orchard. **(A)** Disease incidence on “Golden Delicious” in 2018, **(B)** Disease incidence on “York” in 2018, **(C)** Disease incidence on “Golden Delicious” in 2019, **(D)** Disease incidence on “Rome” in 2019, **(E)** Disease incidence on “Empire” in 2019.

In 2018 on “Golden Delicious,” FireWall^TM^ was the most effective treatment with only 11% of treated clusters developing disease, while the mock treatment showed 89% disease incidence (*p*-value 0.0028 ANOVA, Tukey HDS). No significant differences were observed between BAV2934 with 48%, BlightBan^TM^ with 63%, and BAV3296 with 70% disease incidence, compared to the mock treatment (Figure 4A and Supplementary Table S5).

On ‘York’, the mock treatment showed 76.5% disease incidence. FireWall^TM^ and BAV2934 had 0 and 11.5% disease incidence, respectively, both significantly lower compared to the mock treatment and compared to BlightBan^TM^ with 55.5% disease incidence (*p*-value 0.0016 ANOVA, Tukey HDS). Also BAV3296 with 32.5% disease incidence showed significant control compared to the mock treatment, but not compared to BlightBan^TM^. No significant difference was observed between BlightBan^TM^ and the mock treatment (Figure 4B and Supplementary Table S5).

In 2019, apple blossoms of the varieties “Golden Delicious,” “Empire” and “Rome” were pre-treated as in 2018, but one of the BAV2934 UV-generated mutants that had shown no inhibitory activity *in vitro* was also included. On “Golden Delicious,” although no significant differences were observed among pre-treatments (*p*-value 0.3152 ANOVA, Tukey HDS), FireWall^TM^ was the best inhibitor of fire blight with 30% disease incidence. It was followed by BAV2934 with 55%, the BAV2934 mutant with 65%, BAV3296 with 70%, and BlightBan^TM^ with 80% disease incidence. The mock treatment gave 80% disease incidence (Figure 4C and Supplementary Table S5).

On “Rome,” blossoms pre-treated with BAV3296 had 15% disease incidence, while the FireWall^TM^ and BAV2934 UV-mutant pre-treatment each had 20% disease incidence. These pre-treatments were significantly different compared to the pre-treatment with BlightBan^TM^, which showed 65% disease incidence, and with the mock treatment, which showed 70% disease incidence (*p*-value 0.0083 ANOVA, Tukey HDS). The BAV2934 treatment with 35% disease incidence was not significantly lower than the mock treatment (Figure 4D and Supplementary Table S5).

On “Empire,” the mock treatment showed only 50% disease incidence. FireWall^TM^ showed 37% disease incidence, followed by the BAV2934 mutant with 43%, BAV2934 with 47%, BAV3296 with 53% disease incidence. BlightBan^TM^ had 80% disease incidence, which was higher than the mock treatment negative control (Figure 4E and Supplementary Table S5). None of the treatments were significantly different compared to mock (*p*-value 0.4420 ANOVA, Tukey HDS).

In summary, BAV2934 and BAV3296 reduced disease incidence compared to a mock treatment in four out of five field experiments and this reduction was significant in two of the experiments for BAV3296 and in one experiment for BAV2934. For most field experiments, the reduction in disease incidence by BAV2934 and BAV3296 was similar to BlightBan^TM^ but not as good as FireWall^TM^. The BAV2934 UV-mutant showed inconsistent control and was not significantly different from the BAV2934 wild-type strain in any of the trials (Supplementary Table S5).

### Whole Genome Sequencing Precisely Identified Rain-Borne Bacteria

The genomes of BAV2934 and BAV3296 were sequenced using Illumina HiSeq technology and assembled. Since NCBI does not provide strain identification using genome sequences as query, we used the “Identify using a genome sequence” function at the LINbase web service at linbase.org. LINbase identifies strains as members of genome similarity groups, such as species and intraspecific taxa, based on average nucleotide identity (ANI) (Tian et al., 2019). BAV2934 was identified as *Pantoea agglomerans* and BAV3296 as *P. ananatis*. For BAV2934, we also generated 27 Gb of long reads using the Oxford Nanopore Technologies MinION^TM^ sequencer and carried out a hybrid genome assembly of both the short Illumina and long MinION^TM^ reads, which allowed us to obtain a closed genome of 4,003,977 bp and four circular contigs of 528,933 bp, 205,248 bp, 203,868 bp, and 2,968 bp, respectively. These four contigs probably represent plasmids. Genome sequences were submitted to GenBank with accession numbers GCA_009765475.1 (BAV2934) and GCA_009765415.1 (BAV3296).

### A Combination of Biosynthetic Gene Clusters (BGCs) Prediction and UV-Mutagenesis Identified the Genetic Basis of Antibiosis in *P. agglomerans* BAV2934

To identify putative genes at the basis of the inhibitory activity of BAV2934 against *E. amylovora*, the genome was annotated using Prokka (Seemann, 2014) and biosynthetic gene clusters (BGCs) were predicted using antiSMASH (Medema et al., 2011) (Supplementary Table S6). Eight BGCs were identified that range from 11,391 to 59,810 bp and were predicted to produce a series of different products (Table 3). To identify if any BGC was involved in the inhibitory activity, a UV-mutant screen was performed in parallel. After UV treatment, 1099 colonies of BAV2934 were screened for loss of inhibition *in vitro* and five mutants were identified. DNA of the five mutants was pooled and sequenced on the Illumina platform. 21,742,895 reads of a total length of 7.3 Gb were obtained and mapped against the annotated BAV2934 genome to identify non-synonyms mutations that could explain the loss of the inhibitory activity. Supplementary Table S6 lists the 17 non-synonymous mutations that were found. Gene NOOGOKNH_04505, annotated as a dimodular non-ribosomal peptide synthase, was one of them. Using BLAST, it was identified as part of a phenazine antibiotic D-alanylgriseoluteic acid synthesis gene cluster in *Erwinia herbicola* Eh1087 (Giddens et al., 2002). Among the BGCs predicted in BAV2934 by antiSMASH (Table 3), a phenazine gene cluster was identified in contig 4, and although not in this BGC, the mutation was 1,843 bp downstream of the phenazine cluster (Supplementary Figure S3) and may contribute to production of the compound. This mutation was confirmed by PCR and Sanger sequencing in one of the five mutants; the gene could not be amplified from the other four mutants. Since, based on genome sequencing, the NOOGOKNH_04505 gene is located on the 203,868 bp-long contig in the BAV2934 genome assembly, the lack of amplification may have been due to plasmid loss. In fact, genome coverage data of the reads derived from the sequencing of the five mutant-pool showed that the entire contig was present in the pool only at 1/4th of the average coverage of the main chromosome and PCR with gene-specific primers of chromosomal genes and putative plasmid-encoded genes confirmed the absence of the 203,868 bp-long plasmid in these four mutants (Supplementary Figure S2).

**TABLE 3:** Biosynthetic gene clusters predicted in BAV2934 (GCA_009765475.1) by antiSMASH (Medema et al., 2011).

Contig	Predicted product	BGC coordinates	BGC size	Most similar known cluster	Similarity
1	Arylpolyene,	2,541,579–2,601,389	59,810	APE Ec	78%
	Homoserine lactone				
1	Thiopeptide	2,667,346–2,693,602	26,256	O-antigen	14%
1	Homoserine lactone	3,464,471–3,485,109	20,638	–	
1	NRPS	3,575,053–3,627,116	52,063	Amonabactin	57%
2	Siderophore	158,889–171,243	12,354	Desferrioxamine	100%
2	Terpene	345,771–369,332	23,561	Carotenoid	100%
3	Bacteriocin	104,879–116,270	11,391	–	
4	Phenazine	78,916–99,392	20,476	Pyocyanine	57%

## Discussion

Efficacy of BCAs in the field, in particular when applied to aerial plant surfaces, is dependent on weather conditions during and after application because BCAs need to colonize and survive on plants surfaces in order to efficiently inhibit pathogens (Johnson et al., 2000). In this study, we hypothesized that precipitation may be a promising source of BCAs for aerial plant surfaces, in particular against the fire blight pathogen *E. amylovora* on apple blossoms, since bacteria that are ubiquitous in the atmosphere and precipitation (Polymenakou, 2012) can be expected to be adapted to some of the same environmental stresses to which BCAs are exposed, such as dramatic changes in temperature, humidity, and UV radiation (Lindow, 1991). Additionally, as part of a separate study (Failor et al., 2017), we had isolated bacteria from rain that were members of the genera *Pseudomonas*, *Pantoea*, and *Bacillus*, which include common plant-associated species, some of which are already used in commercial BCAs (Matyjaszczyk, 2015).

As a first step, we screened 254 rain-isolated bacteria for inhibition of *E. amylovora in vitro*. We excluded bacteria with confirmed ice nucleation activity from this screen because they could worsen frost damage when used during weather conditions favorable for frost formation (Lindow, 1983). Nine out of the 254 tested bacteria (3.5%) showed a measurable inhibitory effect against *E. amylovora*. Two isolates, identified as members of the genus *Pantoea*, maintained their efficacy against *E. amylovora* even when pathogen concentrations were increased, while the other isolates failed under these conditions. Our use of precipitation as a source of potential BCAs is unconventional, as traditionally plant surfaces and soil are the most commonly utilized sources for BCAs isolation. Gerami et al. (2013) found that almost 50% of 120 tested epiphytic bacterial strains, isolated from blossoms, leaves and shoots of pome-fruit and stone-fruit trees, inhibited *E. amylovora in vitro* but only 4 isolates worked efficiently in both *in vitro* and *in planta* assays. Sharifazizi et al. (2017) found that 45% of 22 pear leaf-associated bacterial isolates inhibited *E. amylovora in vitro*. It is challenging to compare our success rate with rain-isolated bacteria with the success rate in these screens using epiphytic bacteria since many factors contribute to the efficiency of bacteria to inhibit pathogens *in vitro*, including: the identity of the pathogen strain, the concentration at which potential BCAs and pathogens are plated, and the type of growth medium used (Dickie and Bell, 1995; Borowicz and Saad Omer, 2000). Therefore, in the absence of a direct comparison with other isolation sources, we cannot conclude if rain harbors a higher or lower proportion of bacteria that effectively inhibit pathogens *in vitro* compared to bacteria isolated from other sources. Nonetheless, since we succeeded in identifying two *Pantoea* isolates that strongly inhibited *E. amylovora in vitro*, further tests to determine their survival rate and their efficiency *in vivo* were warranted.

Since the main motivation behind our study was the expectation that rain-isolated bacteria could persist on plant surfaces despite environmental stresses, we tested survival on both apple branches and blossoms. On apple branches, the rain-isolated strains BAV2934 and BAV3296 showed a very similar survival rate to *E. amylovora* declining approximately 100-fold after 12 days post-inoculation in three separate experiments in November, February, and March, while *E. coli* was already undetectable 4 days post-inoculation. The decline was faster in November and February probably due to colder temperatures, compared to those recorded in March (Supplementary Table S3). Although we did not extend survival assays beyond 12 days, the fact that survival of BAV2934 and BAV3296 was similar to survival of *E. amylovora* on apple branches gives confidence that rain-borne bacteria are able to persist on plant surfaces as we had hypothesized. This result also suggests that winter and spring applications of these potential BCAs should be tested, to determine if such applications could reduce fire blight incidence in the following spring and summer.

When testing survival on apple blossoms, the total bacterial population size on blossoms inoculated with BAV2934 was significantly larger than the population size on non-inoculated blossoms 10 days post-inoculation (Figure 2A). Further, the relative abundance of BAV2934 remained stable, representing over 85% of the total population at 10 days post-inoculation (Figure 2B). This evidence also suggests that BAV2934 is able to robustly colonize and persist on healthy apple blossoms under field conditions. In contrast, Wei et al. (2016) found that the relative abundance of a potential BCA (*Bacillus subtilis*) in strawberry leaves was found to be depleted by 50% 8 days post-inoculation under field conditions. Another study found the population size of a potential *Lactobacillus plantarum* BCA decreased significantly 10 days after inoculation on kiwifruit, strawberry, and *Prunus* leaves, even under stable greenhouse conditions (Daranas et al., 2019). When compared to those BCAs, which were isolated from plants or soil, our rain-borne isolate BAV2934 demonstrated better survival on aerial plant surfaces. However, since differences in bacterial survival rate depend on the type of plant surface (Pujol et al., 2006; Bonaterra et al., 2007) and environmental conditions (Nuclo et al., 1998), we cannot make general conclusions before testing our rain-isolated bacteria in different geographic locations.

Some BCAs have a broad spectrum of inhibition against many plant pathogens (Ishimaru et al., 1988; Mora et al., 2015; Daranas et al., 2019). Here, we observed that our initial 9 rain-isolated bacteria have the ability to suppress a wide range of bacterial, fungal, and oomycetes pathogens *in vitro* (Supplementary Table S2). However, the efficacy of the rain-isolated *Pantoea* and *Bacillus* strains was limited to bacterial pathogens. In contrast, the *Pseudomonas* isolates inhibited bacterial, fungal, and oomycete plant pathogens. This suggests either the production of several antimicrobial compounds (Bender et al., 1999) in these strains or the production of an antibiotic with broad spectrum activity (Ishimaru et al., 1988). The broad spectrum activity against fungi and bacteria observed in the rain-isolated *Pseudomonas* strain agrees with previous reports that *Pseudomonas* isolates produce siderophores with antimicrobial properties in disease-suppressive soils (Kloepper et al., 1980). For example, *P. fluorescens* strain UP61 produces three different antibiotics, including pyrrolnitrin, pyoluteorin and 2,4-diacetylphloroglucinol, involved in the inhibition of fungal, oomycete and bacterial plant pathogen strains (La Fuente et al., 2004). Also, several *Pseudomonas syringae* strains produce syringomicins and syringopeptins that inhibit a broad spectrum of fungi and bacteria, respectively (Bensaci et al., 2011).

The effect of timing of BCA application compared to pathogen inoculation is variable. While (Wilson et al., 1992a) reported a significant control of *E. amylovora* on hawthorn blossoms when biocontrol and pathogen were co-inoculated (Wilson et al., 1992b) found that *Pseudomonas fluorescens* strain A506 effectively protected pear blossoms when blossoms were inoculated with the BCA in advance, but not when blossoms were co-inoculated with the BCA and *E. amylovora*. In detached blossom assays, we decided to apply rain-isolated bacteria 2 days before we applied the pathogen to allow sufficient time for blossom colonization by our putative BCAs. In contrast, in the field experiments, BCA treatments were done only 2 h before *E. amylovora* inoculation because we wanted to inoculate both the BCA and the pathogen when the blossoms were most susceptible. Also, since *E. amylovora* can persist in symptomless infected plant tissue (Crepel and Maes, 2000; Weißhaupt et al., 2016), the pathogen can spread and colonize blossoms as soon as they open. Therefore, testing BCAs by applying them only 2 h before inoculation with the pathogen may represent a more realistic scenario for the use of BCAs in agricultural practice.

In some studies, no relationship was found between *in vitro* antibiosis and *in vivo* bacterial performance (Özaktan et al., 1999; Gerami et al., 2013). Our rain-isolated bacteria were initially tested for antibiosis in a dual culture assay against *E. amylovora* (Table 2) with the goal of finding potential new secondary metabolites in further experiments. Then, the best inhibitors of *E. amylovora in vitro* were tested for their ability to suppress fire blight *in planta*. Published assays for testing the efficiency of BCAs in controlling fire blight on detached blossoms only rate disease incidence on whole blossoms (Bonaterra et al., 2007; Roselló et al., 2013). We decided to measure the pathogen population size separately in the receptacle and the pedicel and to measure the necrotic portion of the blossom pedicel. Our reasoning for evaluating the pedicel separately from the receptacle was that the ability of a BCA to reduce symptoms and pathogen population size should be a measure of its ability to interfere with pathogen colonization and migration into the rest of the apple tree. Using this experimental setup, we found that strains with the best *in vitro* performance had *in vivo* performance similar to the commercial BCA BlightBan^TM^ A506, but were not as efficient as the streptomycin product FireWall^TM^.

We observed inconsistent results when the rain-isolated bacteria were inoculated on attached blossoms in an apple orchard (Figure 4). BAV2934 significantly reduced disease incidence compared to the mock treatment in only one out of five trials, while BAV3296 significantly reduced disease incidence in only two out of five trials. In addition, BAV2934 or BAV3296 only performed significantly better than the commercial BCA BlightBan^TM^ in one trial each. The streptomycin product FireWall^TM^ generally performed the best in field trials, significantly reducing disease incidence in three out of five trials. We expected the BAV2934 mutant, which had lost activity in the *in vitro* assays, to perform poorer than the wild-type BAV2934 on attached blossoms, but we could not observe any significant differences between the two. Some of the non-significant differences we observed between treatments may have simply been due to small sample sizes (20 to 30 clusters per treatment per trial), but such variability is generally in line with previous studies using other BCAs. For example (Sundin et al., 2009), found that BCA applications were inefficient and highly variable in the control of fire blight in the field in Michigan, New York, and Virginia. However, it is possible that better and more consistent results could have been obtained if BAV2934, BAV3296, and BlightBan^TM^ had been applied more in advance of pathogen inoculation, allowing BCA organisms more time to colonize the blossoms. BCA variability may also be related to inoculum preparation (Stockwell et al., 1998) showed that lyophilized cells of *P. fluorescens* A506 and *P. agglomerans* C9-1R established better on apple blossoms than inoculum prepared from fresh bacterial cells harvested directly from solid agar medium. Özaktan et al. (1999) tested several formulations of *P. agglomerans* where talc-based formulations showed better control of fire blight on pear blossoms than lyophilized and whey-based treatments. Further adaptive strategies, including osmoadaptation and acidic conditions, have also been tested as means to increase bacterial survival and fire blight control in both controlled laboratory and field conditions (Bonaterra et al., 2007; Daranas et al., 2018). In our study, bacterial cells were harvested from agar medium and used immediately to inoculate apple blossoms in our laboratory and field trials. Therefore, it may be possible to improve the control efficiency of the rain-isolated bacteria by optimizing their formulation. Additional trials in other geographic locations using alternative formulations will be necessary to determine if our rain-isolated BCAs can provide more efficient fire blight control under field conditions than currently available BCA products.

It is possible that temperatures following application and during incubation affected the control demonstrated in the orchard (Figure 4 and Supplementary Table S3). In 2019, control was generally least effective on the cultivar “Empire” and this was true for all biocontrol organisms and by FireWall^TM^. Post-inoculation temperatures were also coolest on this cultivar: 10.9°C during the first 8 days after application and 12.6°C during the 16-day period until data collection. Control was generally better on cultivars “Golden Delicious” and “Rome” in 2019, and post-inoculation temperatures for these cultivars were warmer with 16.6°C during the first 8 days after application and 17.1°C during the 12-day period until data collection. Despite inconsistent results across cultivars, relative percent control appears to be greater on “Golden Delicious” and “Rome” because fire blight infection on the MgSO_4_ control was greater under these warmer conditions. However, varietal differences may also be a factor, as control was greater on “Rome” than on “Golden Delicious” although both cultivars were treated and inoculated on the same day in 2019.

*Pantoea* species have been shown to produce different antibiotic compounds (Wright et al., 2001). In this study we identified a BGC that is predicted to produce a phenazine by *P. agglomerans* isolate BAV2934, and this molecule has been shown to be required for inhibition of *E. amylovora in vitro* by Giddens et al. (2002). The locus identified by UV mutagenesis is likely to contribute to the phenazine production, since the gene is in close proximity to the predicted BGC, in the same polarity, and because Dimodular NRPS are known to contribute to assist in the production of antibiotics (Felnagle et al., 2008). Phenazines are also produced by many other bacteria including: *Pseudomonas* spp. (Thomashow and Weller, 1988), *Streptomyces* spp. (Karnetová et al., 1983), and *P. agglomerans* (Giddens et al., 2002). However, in our rain-isolated bacteria, which included fluorescent *Pseudomonas* and *Pantoea* isolates, we only identified a phenazine cluster gene in BAV2934.

Due to the extensive genetic diversity found within many bacterial species, some species can include both beneficial and pathogenic bacteria. For example, while some *P. agglomerans* strains are commercialized as BCAs, others are known plant pathogens or recognized opportunistic human pathogens, isolated from wounds (Cruz et al., 2007; Lee et al., 2010; Smits et al., 2015). *P. ananatis* also includes both plant pathogens (De Baere et al., 2004; Coutinho and Venter, 2009) and BCAs (Wu et al., 2016). Unfortunately, it is not well known for either *P. agglomerans* or *P. ananatis* if the same strains can be both beneficial and pathogenic, or if some strains are pathogenic, while others are beneficial. The taxonomy within the *Pantoea* genus is also rapidly changing, which leads to another complication in that some *Pantoea* isolates reported as members of a certain *Pantoea* species have been incorrectly identified (Rezzonico et al., 2009). The LINbase web service (Tian et al., 2019) represents a practical tool to improve precise genome-based identification of BCAs, since it allows classification and identification of bacteria at the genus-, species-, and intraspecies ranks. However, precise genome-based prediction of pathogenicity and other phenotypes for strains belonging to a certain species only becomes possible after careful phenotypic characterization of many reference strains within that species. Because this foundational work has not been done yet with either *P. agglomerans* or *P. ananatis*, LINbase was useful in identifying BAV2934 and BAV3296 as members of these species, but it was impossible to infer their safety as BCAs.

In summary, we have found that rain serves as a reservoir of bacteria that suppress growth of *E. amylovora* and other plant pathogens *in vitro*. Two of the bacteria we isolated were shown to control fire blight on detached blossoms, to survive similarly to *E. amylovora* on apple branches and blossoms, and, although inconsistently, to control fire blight in the field. Using a combination of genomics and UV-mutagenesis, we also determined the likely mechanism of antibiosis in one of the isolates. While it was straightforward to identify potential BCAs to the species-level using genomics, a database with more thorough phenotypic characterization of strains in regard to pathogenicity on plants and humans will be necessary to infer safety of BCAs based on their genome sequences alone. Therefore, additional field tests, and formulations and safety tests will need to be performed before making a conclusive determination of the potential of the identified rain-borne bacteria as commercial BCAs.

## Data Availability Statement

Genome sequences were submitted to GenBank and were assigned accession numbers GCA_009765475.1 (BAV2934) and GCA_009765415.1 (BAV3296).

## Author Contributions

MM conducted most of the experiments with contributions from VB and KH. LT contributed to the bioinformatics analysis. HW analyzed genomes for prediction of the biosynthetic gene clusters. BV, KY, and SM developed the overall project. MM and BV wrote the manuscript with input from the other authors.

## Conflict of Interest

LINbase uses the trademarks Life Identification Number^®^ and LIN^®^, which are registered by this Genomic Life, Inc. BV reports in accordance with Virginia Tech policies and procedures and his ethical obligation as researcher that he has a financial interest in this Genomic Life, Inc. Therefore, his financial interests may be affected by the research reported in this manuscript. He has disclosed those interests fully to Virginia Tech, and he has in place an approved plan for managing any potential conflicts arising from this relationship. The remaining authors declare that the research was conducted in the absence of any commercial or financial relationships that could be construed as a potential conflict of interest.

## Supplementary Material

FIGURE S1 Bacterial survival rate of rain-isolated BAV2934, BAV3296 and *E. amylovora* BAV5616 on apple branches under environmental conditions in **(A)** November 2016 and **(B)** March 2017 under environmental conditions.

FIGURE S2 PCR detection of contig 4 in BAV2934. **(A)** Contig 4 gene NOOGOKNH_04505, **(B)** Contig 4 gene NOOGOKNH_04435, **(C)** Contig 4 gene NOOGOKNH_04535, **(D)** Contig 4 gene NOOGOKNH_04609, **(E)** Chromosomal gene NOOGOKNH_2217, and **(F)** Chromosomal gene NOOGOKNH_02219.

FIGURE S3 Identified BGC predicted to encode a phenazine compound. The red dashed box indicates the gene cluster identified by antiSMASH. The black vertical line indicates the location of the base pair mutation. JGI IMG base pair coordinates are indicated.

TABLE S1 List of rain-isolated bacteria tested in the initial inhibition assay.

TABLE S2 Antagonistic activity of rain-isolated bacteria against plant pathogens.

TABLE S3 Bacterial survival assay on apple branches under environmental conditions.

TABLE S4 Weather conditions during field treatments at Kentland Farm, VA.

TABLE S5 Infection (% infected clusters) and control (% reduction compared to MgSO_4_) in the field.

TABLE S6 BAV2934 UV-generated non-synonyms mutations.
