# Supplementary figures and images for: Exploring Rain as Source of Biological Control Agents for Fire Blight on Apple
Source: Front Microbiol. 2020 Feb 14;11:199. doi: 10.3389/fmicb.2020.00199 (PMC7033628; doi:10.3389/fmicb.2020.00199)

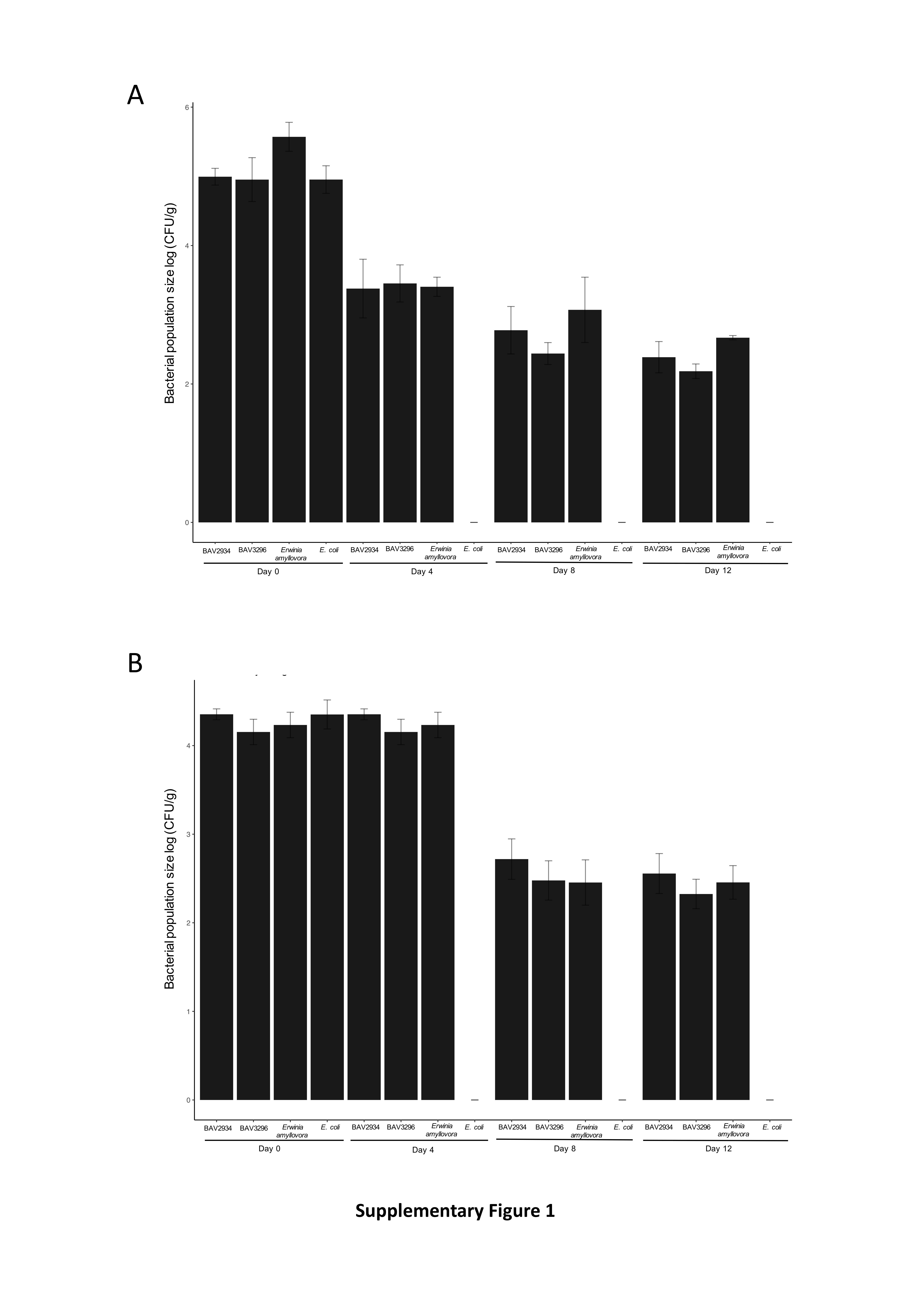

Supplement: FIGURE S1 — Bacterial survival rate of rain-isolated BAV2934, BAV3296 and E. amylovora BAV5616 on apple branches under environmental conditions in (A) November 2016 and (B) March 2017 under environmental conditions. [file Image_1.JPEG]

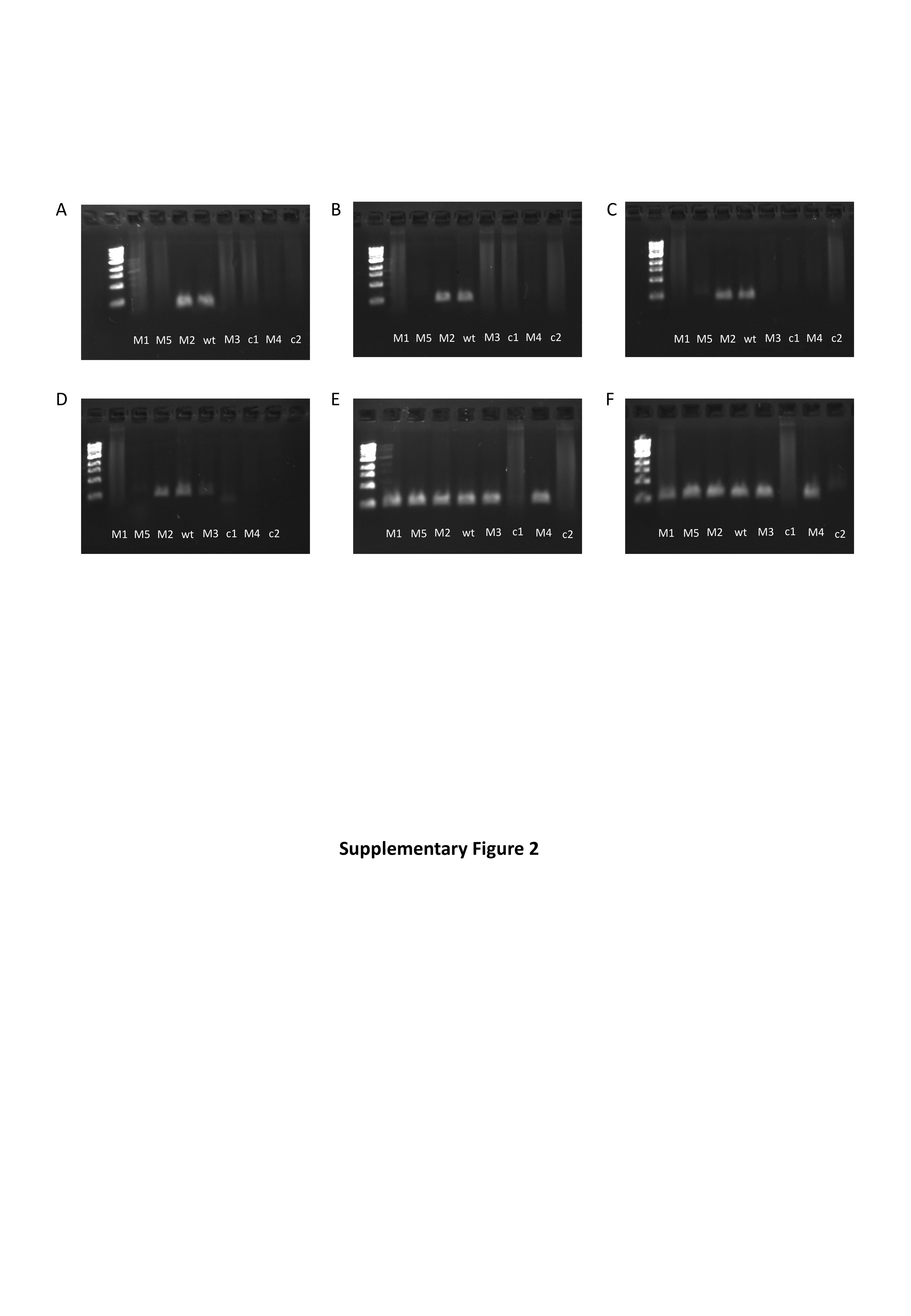

Supplement: FIGURE S2 — PCR detection of contig 4 in BAV2934. (A) Contig 4 gene NOOGOKNH_04505, (B) Contig 4 gene NOOGOKNH_04435, (C) Contig 4 gene NOOGOKNH_04535, (D) Contig 4 gene NOOGOKNH_04609, (E) Chromosomal gene NOOGOKNH_2217, and (F) Chromosomal gene NOOGOKNH_02219. [file Image_2.jpeg]

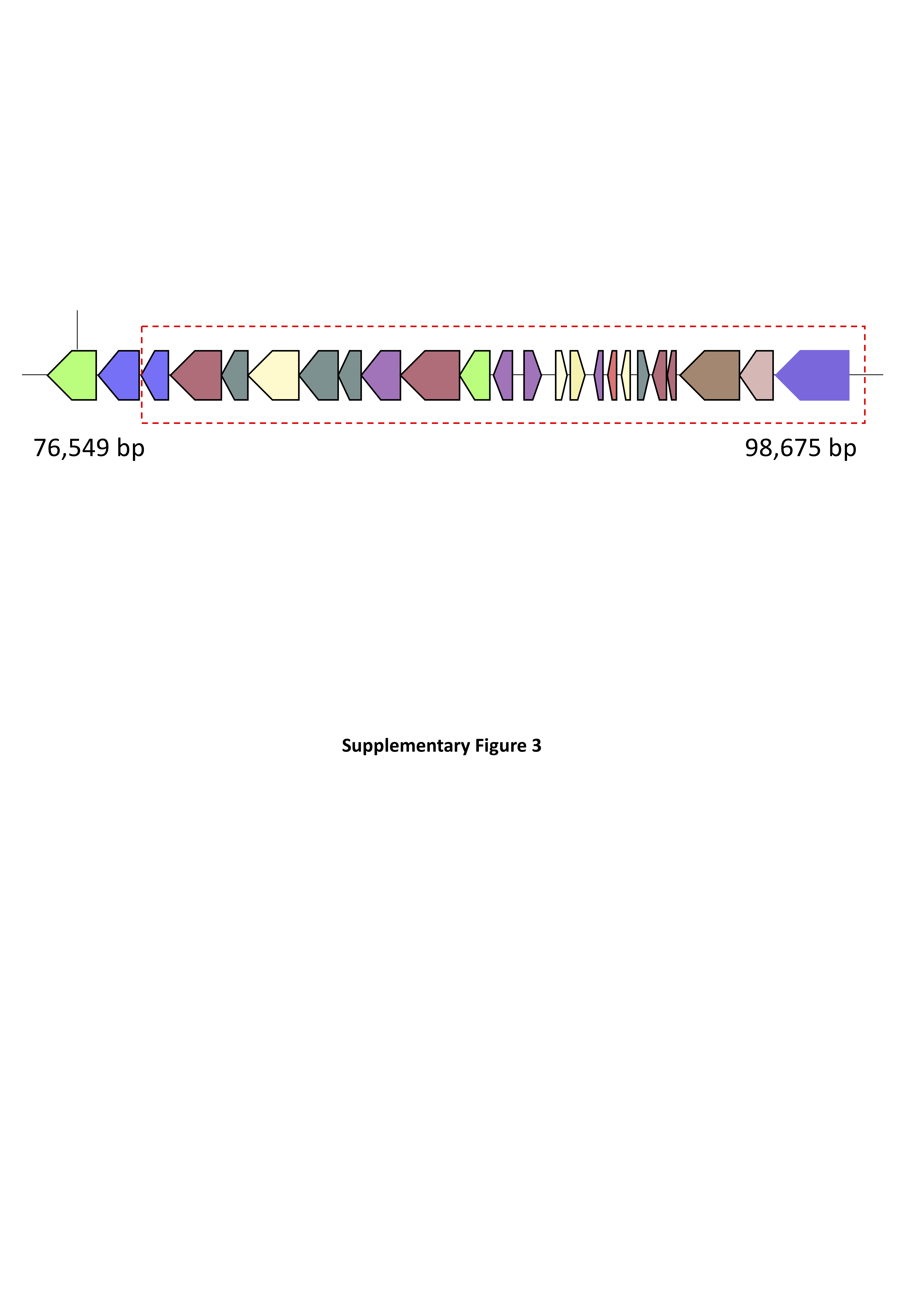

Supplement: FIGURE S3 — Identified BGC predicted to encode a phenazine compound. The red dashed box indicates the gene cluster identified by antiSMASH. The black vertical line indicates the location of the base pair mutation. JGI IMG base pair coordinates are indicated. [file Image_3.jpeg]
